# Gender Disparity in Leadership Positions of General Surgical Societies in North America, Europe, and Oceania

**DOI:** 10.7759/cureus.6285

**Published:** 2019-12-03

**Authors:** Bicong Wu, Nizar Bhulani, Sabeena Jalal, Jeffrey Ding, Faisal Khosa

**Affiliations:** 1 Pathology, University of Washington, Seattle, USA; 2 Surgery, Brigham and Women’s Hospital, Boston, USA; 3 Radiology, Vancouver General Hospital, Vancouver, CAN; 4 Internal Medicine, University of British Columbia, Vancouver, CAN

**Keywords:** surgical societies, h-index, gender disparity, general surgery, academic productivity

## Abstract

Background

Despite the number of female medical-school applicants reaching an all-time high and the increasing number of females in surgical training, males retain an overwhelming majority in senior surgical academic positions and formal leadership positions. This study aims to better understand the extent of and influences for gender disparity in general surgical societies throughout North America, Europe, and Oceania.

Methods

Data collection for this retrospective cross-sectional study took place between June and December 2017. Committee and subcommittee members from the eight selected general surgical societies that met the inclusion criteria (n = 311) were compiled into an Excel spreadsheet in which the data was recorded. Analyzed metrics included university academic ranking, surgical society leadership position, h-index, number of citations, and total publications. SCOPUS database (Elsevier, Amsterdam, Netherlands) was used to generate author metrics, and STATA version 14.0 (StataCorp, College Station, TX) was used for statistical analysis.

Results

Overall, 83.28% of members of the entities we studied were male and 16.72% were females. Males had significantly higher representation than females in all societies (Pearson chi^2 ^= 29.081; p-value = 0.010). Females were underrepresented in all society leadership positions and university academic rankings. Male members had a higher median h-index, more number of citations, and more total publications.

Conclusions

The composition of the general surgical societies included in this study demonstrated significant gender disparity. Female inclusivity initiatives and policies must be initiated to promote greater research productivity and early career opportunities for female surgeons in the specialty of general surgery.

## Introduction

Despite historical gender roles that have legitimized the undervaluation of females, medical school matriculations in recent years have almost attained balanced gender proportions [1]. Females constitute nearly half, if not more, of all medical students in the US, Canada, UK, Australia, and New Zealand [2-5]. However, gender disparity is still present in the leadership of medical schools across North America, Europe, Oceania, and Asia [6]. Factors responsible for the gender disparity in the senior academic ranks of medicine are not fully understood and explored even though female physicians are entering the workforce in comparable numbers to their male counterparts. According to the 2015-2016 report by the Association of American Medical Colleges (AAMC), females account for 21% of full professors, 15% of department chairs, and 16% of medical school deans [7]. Furthermore, only 19.2% of active general surgeons in 2015 were female [8]. The gender disparity in academic surgery is further brought to the fore when we consider that female surgeons only make up 25% of assistant professors, 19% of associate professors, and 10% of full professors [9]. 

Although medicine has come a long way in removing barriers that prevented females from entering the profession and excelling in it, more work is required to achieve gender equity in the profession. Female physicians receive fewer awards and lower monetary compensation compared to their male counterparts [10,11]. Academic productivity including research output is a significant component for academic advancement, but female researchers have greater difficulty attaining primary authorship on their publications [12]. When a double-blind peer review process was tested, a noticeable increase in female authorship was observed [13]. This suggested that the gender disparity in medical academia may be attributed partially to unconscious biases. Gender has no connection with leadership competence, but males are still viewed more favorably than female leaders [14,15]. In Bruce et al.’s (2015) survey of 334 female medical students, residents, and practicing physicians, 87% reported experiencing gender-related discrimination in medical school, 88% in residency, and 91% in practice. However, 40% of all perceived discrimination came from other females [16]. Therefore, both females and males are equally responsible for gender-related discrimination found in the workplace. Because gender disparity is predominantly a social issue, we believed that studying the gender dynamics within the leadership of major general surgical societies was appropriate and timely.

The issue of gender disparity in medicine impedes the improvement of the overall healthcare of females. The specialty of surgery manifests one of the highest degrees of gender imbalance as compared to other specialties [8]. This study sought to identify the extent of and influences for gender disparity within general surgical societies located in North America, Europe, and Oceania. We aimed to draw comparisons between the three regions and to examine the discrepancies between the promotion and career advancement of male and female physicians. We hope that documentation and scientific analysis of the gender disparity found within general surgical societies could generate greater awareness and promote new initiatives to counteract the imbalances at play. 

## Materials and methods

Data collection

**Figure 1:**
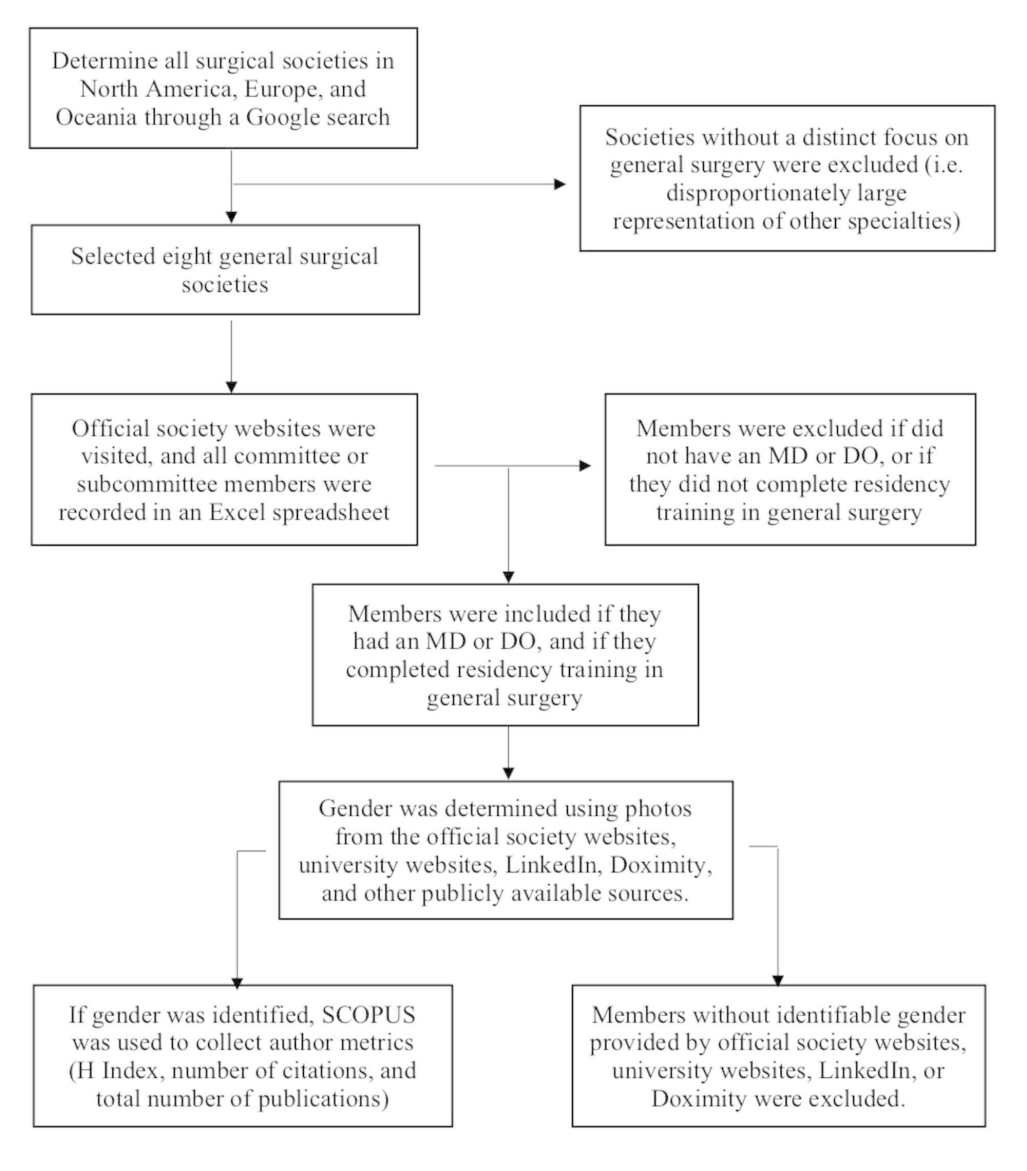
Flow diagram of the data collection process

All the data collected was from publicly available sources, thus institutional review board approval was not required. This study did not involve human subjects and therefore was not registered in a publicly accessible database. Figure 1 is a flow diagram that explains the overall data collection process. The scope of our retrospective cross-sectional study spanned three major geographic entities: North America, Europe, and Oceania. Other parts of the World were not included due to the lack of sufficiently comprehensive English websites for us to extract the pertinent data. Societies were excluded if they were not distinctively focused on general surgery (i.e., societies with broad inclusion of many surgical specialties). These criteria were carefully crafted to include general surgical societies in a reliable and transparent way. Individuals from the general surgical societies from each of the three areas were identified through an in-depth internet search including LinkedIn, Doximity, and University and surgical society websites.

A total of eight societies were selected and compiled into an Excel spreadsheet in which the data were recorded: 1. The American Society of General Surgeons (ASGS); 2. American Surgical Association (ASA); 3. Canadian Association of General Surgeons (CAGS); 4. European Society of Surgery (ESSURG); 5. European Surgical Association (ESA); 6. European Union of Medical Specialists - Section of Surgery and European Board of Surgery (UEMSSURG); 7. General Surgeons Australia (GSA); and 8. New Zealand Association of General Surgeons (NZAGS)

The inclusion criteria included all leadership roles (president, vice president, immediate past president, committee chair, and co-chairs) and committee or subcommittee members in these surgical societies with a medical degree (MD and DO) or equivalent and residency training in general surgery. The committee or subcommittee members were only included if they had a profile on the official university, society, or hospital website. The exclusion criteria included all committee or subcommittee members without an MD or DO, or those not having residency training in general surgery. Individuals without identifiable gender as provided by university websites, LinkedIn, Doximity, and other publicly available sources were excluded from this study. 

Upon identifying all the individuals from the eight general surgical societies who met the inclusion criteria, the SCOPUS database (Elsevier, Amsterdam, Netherlands) was used to generate their author metrics. For retaining consistency, the entire data set was collected by the same researcher between June and December 2017 and rechecked in January and March 2018. The author metrics included h-index, number of citations, and total publications. We chose SCOPUS as our designated tool for collecting the metrics because it provided more comprehensive coverage of articles as compared to other sources [17,18]. An accurate h-index from a reliable database was deemed essential as it not only allowed us to analyze research productivity and impact but also provided a prognostic insight into researchers’ future scientific endeavors [19].

Statistical analysis

STATA version 14.0 (StataCorp, College Station, TX) was used for statistical analysis. Data were tested for normality. Log transformation was done for the continuous variables of h-index, citations, and number of publications, which were initially skewed in distribution. 

At the univariate level, simple linear regression was applied. Each variable was regressed independently with h-index, their assumptions were checked, and their significance was reported. Gender was our primary exposure of interest. Variables that were significant on univariate regression were gender, publications, citations, years since first publication, academic ranks, leadership ranks, and societies. They were selected for inclusion into multivariable linear regression analysis. We checked for multi-collinearity between independent variables and they were assessed using a correlation coefficient. Cramer’s V test was used for one nominal and one ordinal variable, and the Spearman test was used for one continuous variable and one ordinal variable. A correlation of 0.8 was treated as the presence of multi-collinearity. There was no multi-collinearity seen.

The main effects were identified using a stepwise selection strategy and were based on the p-value; we decided to keep a variable in the model or drop it. The multivariable analysis supported the inclusion of gender, citations, publications, years since first publication. Academic rank, leadership rank, and societies were dropped from the model in the preliminary model. The final step involved a check for interaction. Interaction terms were created between each of the main effects in the model. There was a significant interaction between gender and citations and gender and publications. 

The Final Model

 y(x) = β0+ β1(Gender) + β2(Publications) + β3(Citations) + β4(Years since first publication) + β51(Gender*Publications) + β52(Gender*Citations) 

This prediction equation accounted for major variability in the model as adjusted R square was 0.87, F test = 139.54, and p-value = ≤0.001. Compared to males, female faculty had odds of 1.24 times of higher h-index, which indicated that they were more likely to have a comparable h-index to male faculty when adjusting for all other variables. The remaining variability in the model may have been explained by variables such as full-time versus part-time employment, years of employment, and contract versus tenure positions. However, this was beyond the scope of our paper, as we used the data that was available on the internet. The factors which are considered important in academic advancement did not play a role according to our results. 

## Results

**Figure 2:**
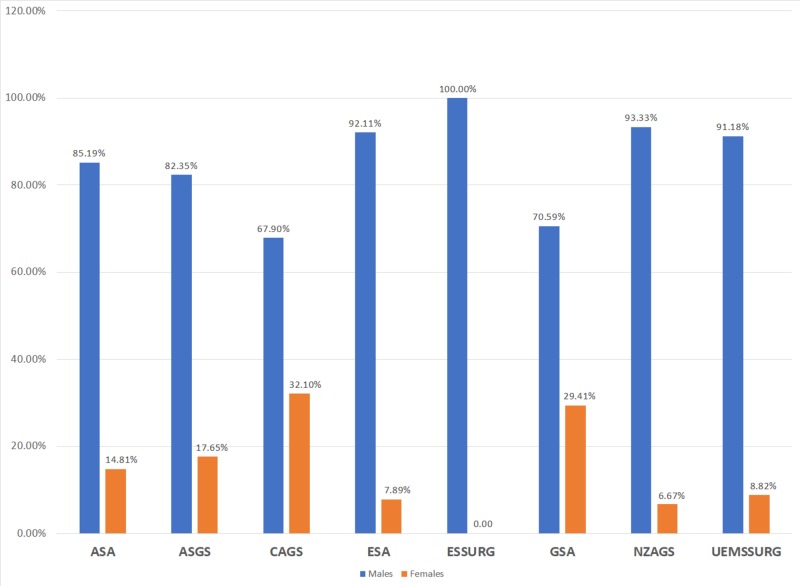
Overall gender proportions in general surgical societies in North America, Europe, and Oceania The blue and red bars represent the male and female proportions, respectively. ASA: American Surgical Association; ASGS: American Society of General Surgeons; CAGS: Canadian Association of General Surgeons; ESA: European Surgical Association; ESSURG: European Society of Surgery; GSA: General Surgeons Australia; NZAGS: New Zealand Association of General Surgeons; UEMSSURG: European Union of Medical Specialists - Section of Surgery and European Board of Surgery

A total of 311 society committee members met the inclusion criteria of this study; 259 (83.28%) were male and 52 (16.72%) were female. Figure 2 illustrates the gender proportions within the eight general surgical societies. The maximum number of females was seen in CAGS (26), whereas ESSURG had no females. Males had significantly higher representation than females in all societies (Pearson chi^2 ^= 29.081; p-value = 0.010). Gender imbalances were observed in both society leadership positions and university academic ranks (Table 1). Females were significantly underrepresented across all levels of university academic rank (Pearson chi^2 ^= 16.15; p-value = 0.013). Although overall underrepresented, there was no significant difference in the number of females in leadership positions across different surgical societies (Pearson chi^2 ^= 4.5464; p-value = 0.805).

**Table 1:** Male and female proportions of leadership positions and academic ranks

		Male	Female
Society leadership position			
	First-in-command	44 (16.99%)	8 (15.69%)
	Second-in-command	9 (3.47%)	4 (7.84%)
	Previous first-in-command	8 (3.09%)	3 (5.88%)
	Member	152 (58.69%)	30 (58.82%)
	Other	46 (17.76%)	6 (11.76%)
	Total	259 (100%)	51 (100%)
University academic rank			
	General faculty	58 (26.36%)	23 (47.92%)
	Assistant professor	10 (4.55%)	5 (10.42%)
	Associate professor	30 (13.64%)	6 (12.5%)
	Professor	122 (55.45%)	14 (29.17%)
	Total	220 (100%)	48 (100%)

Overall, males had a median (range) h-index of 20 (range: 0-101), whereas females had a median of 12 (range: 0-59). Table 2 lays out the h-index distribution of each surgical society. Applying Mann Whitney U test, we noted a significant difference in the median h-index of males and females (Z = 3.061; p-value = 0.0022). The median (range) number of citations for males was 1,479 (range: 0-41,902), whereas females had a median number of citations of 547 (range: 0-16250). Males had a median number of publications of 90 (range: 2-1,230), whereas females had a median number of 27 (range: 2-251). Table 3 includes the median number of citations and publications for males and females within each surgical society. There was a significant difference in the number of overall citations between males and females (Z = 2.976; p-value = 0.0029), and in the number of overall publications between males and females (Z = 3.95; p-value = 0.0001). Table 4 lists the overall statistics of the data analyzed. 

**Table 2:** Male and female h-index distributions across different surgical societies *Values in parenthesis represent ranges ASA: American Surgical Association; ASGS: American Society of General Surgeons; CAGS: Canadian Association of General Surgeons; ESA: European Surgical Association; ESSURG: European Society of Surgery; GSA: General Surgeons Australia; NZAGS: New Zealand Association of General Surgeons; UEMSSURG: European Union of Medical Specialists - Section of Surgery and European Board of Surgery

		Median	
		Males	Females
ASA		41 (7-101)*	34.5 (19-59)*
ASGS		3.5 (2-20)*	12 (7-17)*
CAGS		12.5 (0-43)*	3 (0-20)*
ESA		36 (5-70)*	22 (1-43)*
ESSURG		16 (1-32)*	-
GSA		5 (1-8)*	2 (1-10)*
NZAGS		2 (1-16)*	6 (only value)*
UEMSSURG		19 (1-62)*	24.5 (14-35)*

 

**Table 3:** Median number of citations and publications across surgical societies by gender *Values in parenthesis represent ranges ASA: American Surgical Association; ASGS: American Society of General Surgeons; CAGS: Canadian Association of General Surgeons; ESA: European Surgical Association; ESSURG: European Society of Surgery; GSA: General Surgeons Australia; NZAGS: New Zealand Association of General Surgeons; UEMSSURG: European Union of Medical Specialists - Section of Surgery and European Board of Surgery

	Citations		Publications	
	Male	Female	Male	Female
ASA	5,749.5 (231 -41,902)*	4,463 (1,347-16,250)*	204 (22-1,230)*	166.5 (62-197)*
ASGS	81.5 (48-1,015)*	786 (280-1,292)*	4 (2-90)*	48.5 (20-77)*
CAGS	493.5 (0-10,456)*	44 (0-1,762)*	32 (2-269)*	6 (2-64)*
ESA	5,413 (126-16,715)	4,400 (4-8,796)*	284 (11-524)*	126.5 (2-251)*
ESSURG	813 (5-3,543)*	-	69 (6-224)*	-
GSA	118.5 (3-864)*	66 (2-846)*	9 (2-36)*	4 (3-25)*
NZAGS	49 (0-779)*	104 (only value)	4.5 (2-38)*	10 (only value)
UEMSSURG	1,287 (1-22,464)*	1,781.5 (600-4,357)*	76 (2-377)*	88 (31-117)*

**Table 4:** Data statistics CI: confidence interval

Variables	bCoef.	Standard deviation	t	P-value	95% CI	
Gender	.215	1.29	2.47	0.04	2.343	2.773
Leadership position						
1	2.089	1.24	1.67	0.096	-.373	4.552
2	4.063	2.35	1.72	0.087	-.588	8.716
3	-.175	2.57	-0.07	0.946	-5.24	4.89
4	.597	1.25	0.48	0.633	-1.86	3.064
Academic rank						
1	2.684	2.14	1.25	0.213	-1.549	6.91
2	6.215	1.60	3.88	0.000	3.05	9.374
3	10.495	1.44	7.24	0.000	7.639	13.35
Citations	.0021	.00014	15.23	0.000	.0018	.0024
Publications	.0166	.0057	2.89	0.004	.0052	.0279
Years since first publication	.128	.048	2.66	0.009	.0330	.2233

## Discussion

Gender disparity has been documented in almost all academic medical specialties and subspecialties [20-23]. Even in obstetrics and gynecology, where females make up 85% of the residents in the US, females only hold 21-35% of the departmental academic leadership positions [21,24]. The issue of gender imbalance in academic surgery has also been well documented in previous studies [9,16,25]. Our findings revealed significant gender disparity in the leadership and committee memberships of general surgical societies of North America, Europe, and Oceania. The underrepresentation of female members was consistent across all eight general surgical societies with female proportions ranging from lowest of 0% (ESSURG) to a peak of 32.10% (CAGS). Impediments in the advancement of females in academic surgery may similarly manifest in general surgical societies in a similar capacity. Compared to males, female faculty in the general surgical societies had odds of 1.24 times of higher h-index when adjusting for all other variables. In comparison, Battaglia et al. (2018) noted that this odds ratio was 1.58 for the field of general surgery in North America [23]. In alignment with a 2015 report that noted 19.2% as the female representation of all practicing general surgeons, this study observed a comparable female proportion of 16.72% among the members that met the inclusion criteria (n = 311) [8]. 

Greater gender gaps were also observed at higher university academic ranks. Among the 48 female members who held an academic position, 23 (47.92%) were general faculty and 14 (29.17%) were full professors. Not only were there significantly more male members who held an academic position than females (220 males versus 48 females), the number of males with the ranks of general faculty and full professorship was 58 (26.36%) and 122 (55.45%), respectively. The most common academic position among male and female members was full professor (55.45%) and general faculty (47.92%), respectively. We noted the greatest degree of gender imbalance within the rank of full professor as only 10.29% were female (14 females versus 122 males). The underrepresentation of females was least pronounced in the rank of assistant professor with a female proportion of 33.33% (5 females versus 10 males). In terms of leadership positions within the general surgical societies, 15.38% of the current first-in-command were female (8 females versus 44 males). Males outnumbered female members in all levels of leadership. The male dominance and increasing gender gap at higher academic ranks observed in this study were consistent with the gender disparity previously reported in other medical societies such as radiology and neurosurgery [20,22]. To further understand the observed disparities, we compared h-index, number of citations, and total publications between male and female members across the societies. Overall, males had a higher median h-index, more number of citations, and more total publications than their female counterparts. Using the Mann Whitney U test, we determined that the male and female differences in median values for all three of the studied author metrics were significant. 

Research productivity is a decisive factor for advancement in academic medicine. Studies have shown that female physicians publish less, secure fewer first authorship publications, and apply for fewer grants [16,26,27]. These elements may curtail research output and perpetuate the disproportionately low representation of females in senior levels of medical academia. Recruitment, promotion, and retention of female surgeons must be adequately addressed to rectify the gender imbalance in academic medicine and, subsequently, within the general surgical societies. Female medical students were reported to express a more negative understanding of the surgery profession and its consequences on personal life compared to practicing female surgeons who had a more positive outlook [28]. However, practicing female surgeons were more likely than their male counterparts to perceive that family commitments (e.g., raising a child) delayed career advancement [29]. Especially during the childbearing period, early-career academic productivity of female physicians may be slow due to the additional family and home responsibilities and pressures [30]. Furthermore, pertaining to the issue of retainment, female assistant professors in academic general surgery most commonly contemplated leaving academia [25]. On the whole, female inclusivity policies must be promoted to bridge the gender gap and to mitigate the historical male-dominant gender ratios within academic surgery.

Limitations

Data collection depended on publicly available material including those from surgery society and institutional websites. Time-stamps were usually unavailable on these web pages, and therefore the information extracted may have been out of date during our time of access. There is also the issue of consistency and compliance because some institutions may require faculty members to submit their curriculum vitae or profile information. The other potential limitation involved the SCOPUS database and the collection of author metrics. The database was unable to provide accurate metrics if an individual changed their name, thus resulting in a misidentification error as two authors.

## Conclusions

Gender disparity definitely exists within general surgical societies in North America, Europe, and Oceania. Male committee members had significantly greater representation than female committee members across all societies. Females were underrepresented in all university academic ranks and society leadership positions. Males had a higher median h-index, more number of citations, and more total publications. In line with similar sentiments articulated by previous gender-disparity studies, targeted female-inclusivity initiatives and policies must be instituted for academic general surgery. More studies are required to further investigate the causes of disparities identified in this study and to help in developing initiatives and policies to promote gender diversity.
